# Episodic Decrease in Temperature Increases *mcy* Gene Transcription and Cellular Microcystin in Continuous Cultures of *Microcystis aeruginosa* PCC 7806

**DOI:** 10.3389/fmicb.2020.601864

**Published:** 2020-12-03

**Authors:** Robbie M. Martin, Mohammad Moniruzzaman, Gwendolyn F. Stark, Eric R. Gann, Dominique S. Derminio, Bofan Wei, Ferdi L. Hellweger, Ameet Pinto, Gregory L. Boyer, Steven W. Wilhelm

**Affiliations:** ^1^Department of Microbiology, The University of Tennessee, Knoxville, Knoxville, TN, United States; ^2^Department of Biological Sciences, Virginia Polytechnic Institute and State University, Blacksburg, VA, United States; ^3^Department of Chemistry, State University of New York College of Environmental Science and Forestry, Syracuse, NY, United States; ^4^Water Quality Engineering, Technical University of Berlin, Berlin, Germany; ^5^Civil and Environmental Engineering, Northeastern University, Boston, MA, United States

**Keywords:** microcystin, temperature, cyanotoxins, cyanobacteria, episodic events

## Abstract

Microcystins produced during harmful cyanobacterial blooms are a public health concern. Although patterns are emerging, the environmental cues that stimulate production of microcystin remain confusing, hindering our ability to predict fluctuations in bloom toxicity. In earlier work, growth at cool temperatures relative to optimum (18°C *vs.* 26°C) was confirmed to increase microcystin quota in batch cultures of *Microcystis aeruginosa* NIES-843. Here, we tested this response in *M. aeruginosa* PCC 7806 using continuous cultures to examine temporal dynamics and using RNA-sequencing to investigate the physiological nature of the response. A temperature reduction from 26 to 19°C increased microcystin quota ∼2-fold, from an average of ∼464 ag μm^–3^ cell volume to ∼891 ag μm^–3^ over a 7–9 d period. Reverting the temperature to 26°C returned the cellular microcystin quota to ∼489 ag μm^–3^. Long periods (31–42 d) at 19°C did not increase or decrease microcystin quota beyond that observed at 7–9 d. Nitrogen concentration had little effect on the overall response. RNA sequencing indicated that the decrease in temperature to 19°C induced a classic cold-stress response in *M. aeruginosa* PCC 7806, but this operated on a different timescale than the increased microcystin production. Microcystin quota showed a strong 48- to 72-h time-lag correlation to *mcy* gene expression, but no correlation to concurrent *mcy* expression. This work confirms an effect of temperature on microcystin quota and extends our understanding of the physiological nature of the response.

## Introduction

Cyanobacterial blooms plague fresh waters across the globe. A recent study confirmed that bloom frequency and duration have increased over the past three decades, a pattern long suspected (Ho et al., 2019). Trends in population, urbanization, land use, and temperature due to climate change all but ensure continued eutrophication of freshwater resources (Pachauri et al., 2014; Shukla et al., 2019; Jenny et al., 2020). Blooms degrade ecosystem services of the lakes they occupy, and the noxious effects of blooms are well recorded (Watson et al., 2016; Huisman et al., 2018). Additionally, the cyanotoxins frequently produced during blooms constitute a potential and growing public health concern (Carmichael and Boyer, 2016).

Of the toxins produced by bloom-forming cyanobacteria, microcystins are the most commonly detected (Loftin et al., 2016; Mantzouki et al., 2018). These compounds act as potent liver toxins in vertebrates (MacKintosh et al., 1990). While the physiological function of microcystin continues to be debated (Omidi et al., 2018), there is growing evidence that microcystin plays a role in moderating damage from oxidative stress by protecting key proteins involved in photosynthesis and carbon fixation (Zilliges et al., 2011; Wei et al., 2016). This picture is complicated by reports that microcystins can interfere with the ability of *Microcystis* to cope with externally induced oxidative stress (Schuurmans et al., 2018). These seemingly inconsistent results suggest a complexity in the function of microcystin that is yet to be fully revealed (Barchewitz et al., 2019).

In a similar vein, the environmental cues that stimulate and constrain the production of microcystin remain puzzling (Wilhelm et al., 2020), although general trends are emerging. For example, it is now accepted that higher nitrogen availability leads to greater microcystin production (Gobler et al., 2016). Nevertheless, even this trend can be at odds with lab studies investigating the influence of nitrogen availability on microcystin quota (Ginn et al., 2010; Pimentel and Giani, 2014; Peng et al., 2018). Again, these seemingly inconsistent results point to a combination of variability between strains and a complex regulation of microcystin biosynthesis. Ultimately, our ignorance regarding the function and regulation of microcystin hinders our ability to predict trends and fluctuations in bloom toxicity.

Previously we demonstrated that seasonally relevant cool temperatures increased cellular microcystin content in batch cultures of *Microcystis aeruginosa* NIES-843 (Peng et al., 2018). This cool-temperature phenotype is consistent with and may help explain field observations in which blooms are more toxic in early spring and less toxic in midsummer (Kardinaal and Visser, 2005). In the lab, this response to cool temperature could serve as a controllable phenotype to investigate the eco-physiological function of microcystin. Yet, key questions involving how this phenotype manifests in other strains and the temporal dynamic and physiologic nature of the response remain unanswered. In this work, we demonstrate, using continuous cultures, that an episodic decrease in temperature increases microcystin production in *M. aeruginosa* PCC 7806 and that this production reverts to previous rates with a return of temperature. Using RNA sequencing, we show that cellular microcystin is time-lag (48 h) correlated to *mcy* expression and that this expression appears to be partitioned from a generalized cold-stress response. Additionally, we show that nitrogen concentration and temperature affect cell size. Together, this work confirms the effect of reduced temperature on microcystin quota and extends our understanding of the physiological nature of this response.

## Materials and Methods

### Strains and Culture Conditions

Non-axenic cultures of *Microcystis aeruginosa* strain PCC 7806 were grown in medium modified from original CT (Watanabe and Ichimura, 1977). Final nitrogen (N) concentrations were either 81, 161, or 323 μM as a mix of Ca(NO_3_)_2_ and KNO_3_ which contributed N at a molar ratio of 1.37:1. Total phosphorus (P) was supplied at 16.3 μM by K_2_HPO_4_. Resulting N:P ratios were ∼5, 10, and 20 for respective N concentrations. Other components of CT were unchanged. The final pH was adjusted to 8.2 using 0.6 mL of 1 M NaOH. Our isolate of PCC 7806 was obtained from the Pasteur Culture Collection over a decade ago. The strain was re-verified before the start of the experiment *via* PCR/Sanger sequencing using cyanobacteria-specific 27F/809R primers (Jungblut et al., 2005).

Chemostats (described below) were kept in illuminated incubators (VWR model 2015-2). Experimental temperature was set to either 19 or 26°C and was measured every 30 min using a Hobo Tidbit TempLogger (OnSet Computer Corporation) that was suspended in a volume of water equal to the culture volume (1 L). Continuous light at a photosynthetic fluence rate of 50 μmol photons m^–2^ s^–1^ was supplied by fluorescent bulbs (GE Ecolux 32W).

### Chemostat System and Construction

Chemostats were constructed using standard 2 L glass media bottles (VWR) as growth vessels. Vessels were sealed with GL45 Diba Omnifit T-series solvent caps (Cole-Parmer) with 4 ports to separately accommodate feed, weir, aeration, and sampling tubes. Rigid 1/8 in OD PTFE tubing extending through the GL45 cap ports provided airtight extensions to which flexible tubing could be securely connected. Masterflex L/S 14 silicon tubing was used for all other plumbing lines. To aerate the vessel, air filtered through a 0.22-μm nylon syringe filter was supplied by a standard aquarium pump (Topfin Air-500) at a flow rate of 0.5 L min^–1^. In addition, this airflow generated the pressurized headspace necessary to expel excess media through the weir tube to the waste vessel. The adjustable-height weir tube was set to maintain vessel volume at 1 L leaving a headspace of 1 L. Standard 2 or 5 L media bottles using 2-port GL45 caps accommodating a feed tube and a filtered air vent were used for culture medium reservoirs. Individual, single-channel, ultra-low-flow peristaltic pumps (Traceable Products #3384) supplied continuous flow of fresh media. Constant stirring with a magnetic stir bar was used to prevent cell sedimentation. Dilution rate (D) was maintained at 0.25 d^–1^ for all experiments reported here. Three independent chemostat vessels were used in this study.

### Sample Collection

Samples were collected from luer-lock sampling ports to measure microcystin content, cell concentration/size, and for RNA extraction. Samples (50 mL) for microcystin quantification were collected on 47-mm glass filters (Advantec MFS GF75, 0.3 μm retention) *via* vacuum filtration, flash-frozen in liquid nitrogen, and stored at −80°C until processing. Samples for RNA (30 mL) were collected on 47-mm diameter polycarbonate filters (Whatman Nucleopore, 1.0 μm pore-size) *via* vacuum filtration, flash-frozen in liquid nitrogen, and stored at −80°C until extraction.

### Predicting Mean Cell Diameter and Determining Culture Biovolume

Cell concentration was determined *via* flow cytometry using a Guava easyCyteHT (Millipore) flow cytometer gating on red fluorescence (a proxy for chlorophyll *a*) and forward scatter (a proxy for size). Mean cell diameter was predicted by a linear regression equation using forward scatter as the explanatory variable. To produce this regression equation, we measured both the forward scatter and the actual mean cell diameter from concurrent samples across a broad range of conditions. Mean cell diameter was measured with a FlowCam 8000 (Yokogawa Fluid Imaging Technologies) using a 20x objective and a FOV50 flowcell. A minimum of ∼5,000 cells was measured per sample using auto-imaging mode. The binary image particle property “area-based diameter” was used as cell diameter (Spaulding, 2014). Mean cell volume was calculated from predicted mean diameter assuming spherical cells. Culture biovolume was calculated as the product of mean cell volume and cell concentration of the culture.

### Ion Chromatography

Two mL of culture were collected and filtered through a 0.22-μm pore-size SFCA syringe filter (Thermo Scientific). The filtrate was stored at 4°C until analysis. Each sample was analyzed for NO_3_^–^ by ion chromatography using a Dionex ICS2100 Ion Chromatography System (Thermo Scientific) following Standard Methods 4110B with chemical suppression (American Public and Health Association, 2017).

### Microcystin Extraction and Quantification

Particulate microcystins were quantified *via* coupled liquid chromatography/mass spectrometry using methods detailed in Boyer (2007, 2020). Glass fiber filters were extracted in 5 mL of 50% methanol containing 1% acetic acid using ultrasound sonication (three 20-s pulses separated by 20-s rests). Samples were clarified by centrifugation at 14,000 × *g* for 10 min at 4°C. The supernatants were filtered through 0.45 μm pore-size nylon syringe filters (Corning, CLS431225) and stored at −20°C until analysis. Reverse-phase HPLC using a Waters 2695 Separations Module coupled to a Waters ZQ4000 mass spectrometer (m/z 500–1250 amu) and a 2996 photodiode array detector (210–400 nm wavelength) was used to screen for molecular ions of 22 common microcystin congeners (RR, dRR, mRR, H4YR, hYR, YR, LR, mLR, zLR. dLR, meLR, AR, FR, WR, LA, dLA, mLA, LL, LY, LW, LF, WR). Separation conditions used an ACE 5 C18, 150 × 3.0 mm column and a 30–70% aqueous acetonitrile gradient containing 0.1% formic acid at a flow rate of 0.3 mL min^–1^. Concentration of individual congeners was quantified using the peak area of the extracted ion relative to standards of microcystin-LR or microcystin-RR (Enzo Life Sciences). This allows quantification of congeners where standards are not available. Detection of congeners was validated by co-occurring presence of the diagnostic UV signature from the ADDA group. Matrix spike experiments indicated that matrix inhibition was less than 1% so individual samples were not corrected for matrix suppression. Individual method detection limits were calculated from sample volumes and from specific instrument detection limits of the day samples were run (Boyer, 2020). The method detection limit for all samples was equal to or less than 0.32 μg L^–1^ (microcystin-LR equivalents). Total microcystin concentrations are reported as the sum of all congeners.

### RNA Extraction, RNA Sequencing, and Gene Expression Analysis

RNA was extracted using the acid phenol/bead beating protocol described in Martin and Wilhelm (2020). Genomic DNA was digested using Turbo DNA-*free* kit (Ambion) following the method described in Martin and Wilhelm (2017). Samples were considered DNA-free if no bands were visible in an agarose gel after 30 cycles of PCR amplification using standard 27F/1522R primers targeting the 16S rRNA gene. Ribosomal RNA was depleted using the Ribominus Transcriptome Isolation Kit for yeast/bacteria (Invitrogen). Total RNA was quantified and checked for quality using an Agilent 2100 Bioanalyzer.

Libraries (cDNA) were prepared at The University of Tennessee Knoxville Genomics Core using the Illumina TruSeq Stranded mRNA Sample Preparation Kit following manufacturer instructions for the LT/LS protocol by adding the depleted RNA to the Fragment/Prime/Finish step of the protocol. Four multiplexed libraries, at a final cDNA concentration of 4 pM with 2% PhiX spiked-in, were sequenced per run on the Illumina MiSeq platform using Version 3 flowcells, generating 75-bp paired-end reads.

Residual ribosomal reads were removed *in silico* using the SortMeRNA algorithm on default settings (Kopylova et al., 2012). RNA-seq analysis was performed using CLC Genomics Workbench (v. 10.1.1). Reads were trimmed for quality using default parameters and mapped against annotated regions of the reference genome *M. aeruginosa* PCC 7806SL (GenBank accession NZ_CPO20771.1, version annotated January 28, 2020) (Zhao et al., 2018). Default parameters for mismatch, insertion, and deletion costs and custom settings of 0.8 for length fraction and 0.95 for similarity fraction were used. Expression values were calculated as transcripts per million (TPM) (Wagner et al., 2012) using only reads that mapped as pairs. Paired reads were counted as a single mapped fragment; reads mapped as broken pairs were ignored. Weighted gene correlation network analysis (WGCNA) (Zhang and Horvath, 2005) was performed as implemented in the R package WCGNA (Langfelder and Horvath, 2008). Genes with an average expression > 2 counts per million were included in this analysis. Expression values were normalized *via* TMM using edgeR (Robinson et al., 2010). A soft-thresholding power of 12 was used, generating a “signed” network with a minimum module size of 30 genes. We used OmicsBox (Götz et al., 2008) to assign Gene Ontology (GO) terms (Ashburner et al., 2000; Gene Ontology Consortium, 2019) to 3,150 of the 4,834 CDS in the PCC 7806SL genome and to conduct enrichment analysis of GO terms associated with genes assigned to WGCNA modules. Sequence libraries are publicly available and can be found in the NCBI Sequence Read Archive under Project Accession # PRJNA650205.

### Calculation of Microcystin Production Rates

Particulate microcystin production rates were calculated using the following formula:

$$\frac{d\,M\,C}{d\,t}=\text{P}\cdot \text{B}-\text{MC}\cdot \text{D}$$

where MC is microcystin concentration (μg L^–1^), P is biovolume-based production rate (μg mm^–3^ d^–1^), B is biovolume concentration (mm^3^ L^–1^), and D is chemostat dilution rate (d^–1^). Mean production rate for a given temperature period was calculated as the mean of daily production rates for that period.

### Statistical Analysis

Statistical calculations were conducted in GraphPad Prism (v. 8.4.2). For repeated-measures (RM) ANOVA, assumption of sphericity was tested in the R statistical environment (v. 3.4.0); the Greenhouse–Geisser correction was applied when sphericity was not met (Greenhouse and Geisser, 1959). *Post hoc* multiple comparisons were adjusted with Tukey’s HSD. A significance level of *p* = 0.05 was used in all analyses.

## Results

To examine the effects of a temperature change on cellular microcystin concentration, we grew *M. aeruginosa* PCC 7806 in continuous cultures at a standard lab temperature of 26°C. Over an experimental period of 21 days, steady-state cultures were first sampled daily for 5 days at 26°C temperatures to establish a baseline. Vessel temperature was then decreased to 19°C and cultures sampled for 9 days. Subsequently, the temperature was returned to 26°C and cultures sampled over an 8-day period during recovery (we note day 16 was missed).

### Nitrogen and Temperature Treatments Induce Cell Size Change

**FIGURE 1:**
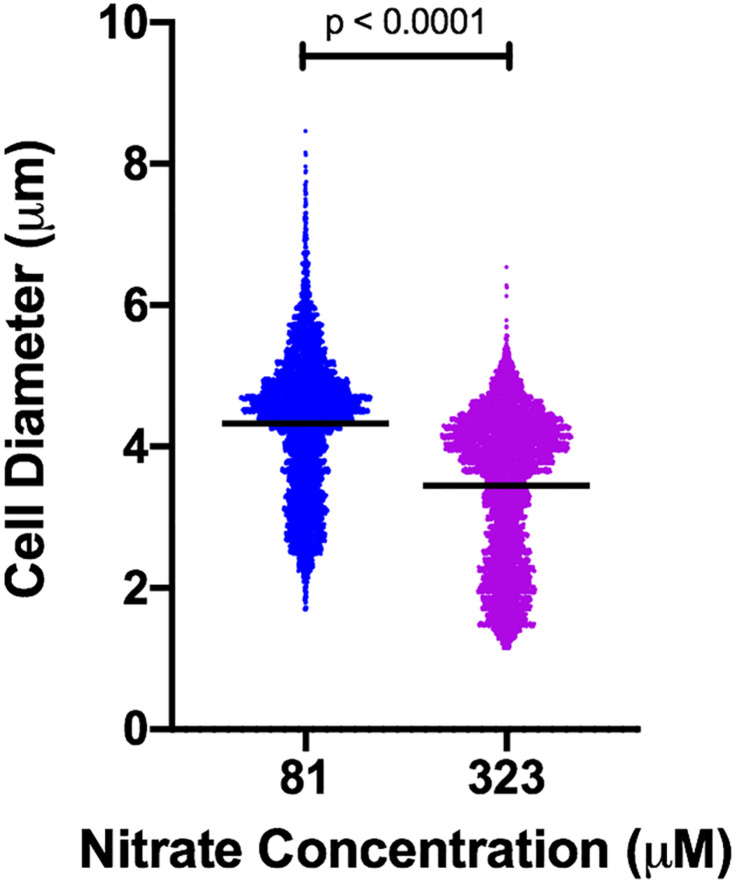
Violin plot of cell diameter in cultures of *M. aeruginosa* PCC 7806 grown in different concentrations of nitrate. Black horizontal bars represent mean diameter. Each measured cell is represented by a plotted point. Width of plot is proportional to frequency of cells at a given diameter. *n* = 4,890 (81 μM); *n* = 5,213 (323 μM). *p*-value from Mann–Whitney test.

**FIGURE 2:**
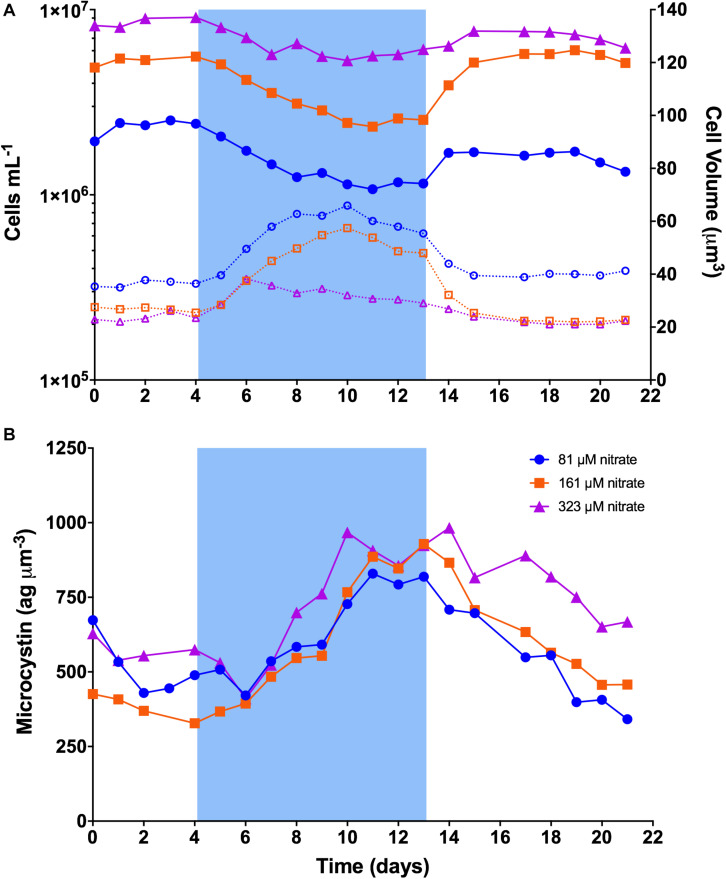
Dynamics of cell concentration, cell volume, and cellular microcystin concentration in continuous cultures of *M. aeruginosa* PCC 7806. Blue shading represents the time period when temperature was set to 19°C. **(A)** Cell concentration and cell volume. Solid lines represent cell concentration on the left *y*-axis. Dashed lines represent cell volume on the right *y*-axis. **(B)** Cellular microcystin concentration.

We observed notable shifts in cell size in response to nitrogen concentration and temperature. These cell size differences were sufficient to influence normalization of microcystin measurements and complicated comparisons of per cell quota. A necessary first step, then, was to address the issue of dynamic cell size. To resolve this, we measured cell diameter across all treatment conditions and related those diameters to mean forward scatter (Supplementary Figure S1). Mean forward scatter described ∼96% of the variation in measured mean cell diameter (*R*^2^ = 0.96) and the slope was significantly non-zero (*p* < 0.0001). Cell diameter increased with decreasing N concentration, resulting in large differences in estimated mean cell volume. For example, the mean cell diameter at steady-state in nitrate concentration of 81 μM was ∼4.33 μm (S.D. 1.01 μm), while for cells growing in 323 μM it was ∼3.45 μm (S.D. 1.05 μm) (Mann–Whitney *p* < 0.0001), representing a 2-fold difference in volume (Figure 1). While the size relationship to nitrogen concentration was consistent, it fluctuated over the course of continuous cultures and was affected strongly by changes in temperature (Figure 2). Given the observed changes in cell size, microcystin measurements were normalized to mean cell volume rather than being reported as a per cell quota. Normalizing this way allows comparison of results between tested conditions.

Chemostat theory predicts that at steady state, concentration of the limiting substrate is relatively constant over a range of dilution rates and is independent of the substrate concentration in the inflowing medium (Herbert et al., 1956). To investigate the effects of nitrogen availability on microcystin production, we empirically tested a range of inflowing nitrate concentrations and selected concentrations that on the higher end (323 μM N) resulted in an excess of nitrate as measured by ion chromatography (and indicating limitation by another nutrient, likely phosphorus), and that on the lower end (81 μM N) clearly resulted in nitrogen-limitation. The third concentration (161 μM N) was selected to be intermediate of these two. The three inflowing nitrate concentrations influenced cellular physiology as evidenced by differences in steady-state cell size (Figure 1) and in chlorophyll *a* fluorescence of individual cells as measured by flow cytometry (Supplementary Figure S2).

### A Temperature Drop Increased Cellular Microcystin Concentration and Cell Size

Actual mean temperatures during the experimental periods were 26.4°C (*SD* = 0.05°C), 19.4°C (*SD* = 0.16°C), and 25.9°C (*SD* = 0.07°C) for the baseline, cold stress, and recovery periods, respectively. A trace of the actual temperature measurements is shown in Supplementary Figure S3. Chemostat vessels equilibrated to within ∼0.3°C of the targeted temperature within ∼6 h of temperature adjustment.

The dynamics of PCC 7806 cultures are illustrated in Figure 2. Steady-state (baseline) cell concentration at 26°C was ∼2.3-, 5.3-, and 8.6 × 10^6^ cells mL^–1^ in 81 μM, 161 μM, and 323 μM N, respectively. After the temperature was decreased to 19°C, growth rates slowed to less than the dilution rate and cell concentration began to decline as cells were washed out of the chemostats (Figure 2A). In 81 and 161 μM N, cell concentration reached a minimum after 7 days at 19°C, dropping to ∼1.1 × 10^6^ mL^–1^ (48% of baseline) and ∼2.3 × 10^6^ mL^–1^ (43% of baseline), respectively. In 323 μM N, cell concentration reached a minimum after 6 days dropping to ∼5.3 × 10^6^ mL^–1^ (62% of baseline). Cell concentration then stabilized and increased slowly to ∼50, 48, and 71% of baseline by the end of the cold stress period for 81, 161, and 323 μM N, respectively. Cell concentrations increased sharply after temperature was returned to 26°C, rebounding to an average of ∼67, 107, and 83% of baseline, respectively. Cell concentration remained relatively constant across the final days of the experiment.

Cell size increased at the onset of cold stress (Figure 2A). Cells in 81 and 161 μM N showed similar trends, increasing to ∼181 and 215% of their respective baseline volumes after 6 days at 19°C. Volumes declined gradually until the temperature returned to 26°C, after which they declined sharply back to baseline volume. Cells in 323 μM N reached peak volume of ∼162% of baseline after 2 days at 19°C, then gradually declined to baseline volumes.

Microcystin content per biovolume showed similar trends among nitrogen concentrations (Figure 2B). While variable across the baseline period, it increased markedly after about 2 days at 19°C, reached a peak ∼7–9 days after onset of cold stress, then declined sharply after temperature was returned to 26°C. On the last day of baseline, mean microcystin content across nitrogen concentrations was 464 ag μm^–3^ (range: 328–574 ag μm^–3^). It increased to ∼891 ag μm^–3^ by the last day of cold stress (range: 819–929 ag μm^–3^), and returned to ∼489 ag μm^–3^ by the final day of recovery (range: 342–668 ag μm^–3^). Means between periods differed (RM-ANOVA *p* = 0.011); baseline and recovery differed from cold stress (Tukey’s *p* = 0.015, *p* = 0.018), but not from each other (Supplementary Figure S4).

Two congeners of microcystin were detected: microcystin-LR and [D-Asp3]-microcystin-LR. This is consistent with previous reports (Tonk et al., 2009; Yeung et al., 2016). The congener profile varied in response to nitrogen concentration and temperature and is illustrated in Supplementary Figure S5.

To statistically test the temporal pattern of microcystin, we determined the slope of the regression line of cellular microcystin content *vs.* time for each nitrogen concentration in each treatment period (Supplementary Figure S6). Within a treatment period, slopes between nitrogen concentrations were similar (Supplementary Figure S6A; baseline *p* = 0.39, cold stress *p* = 0.18, recovery *p* = 0.29). Mean slopes differed between periods (RM-ANOVA *p* = 0.0004); baseline and recovery differed from cold stress (Tukey’s *p* = 0.016, *p* = 0.010) but not from each other (Supplementary Figure S6B). Mean slope during baseline was −26.5 ag μm^–3^ d^–1^ but was not different from zero (one sample *t*-test *p* = 0.13). Mean slope increased to 64.8 ag μm^–3^ d^–1^ during cold stress, and declined to −50.9 ag μm^–3^ d^–1^ across recovery.

If microcystin production is constitutive, it is conceivable that the increasing cellular microcystin content observed during the 19°C period could be due to constitutive production coupled with the reduced growth rate observed during this period. To examine this possibility, we calculated mean microcystin production rates during each of the experimental periods. Across N concentrations, microcystin production (normalized to culture biovolume) averaged 0.11 μg per cubic mm cell biovolume per day (μg mm^–3^ d^–1^) during baseline (range: 0.08–0.14 μg mm^–3^ d^–1^). It increased ∼73% to 0.19 μg mm^–3^ d^–1^ during the period of cold stress (range: 0.17–0.20 μg mm^–3^ d^–1^), and returned to 0.11 μg mm^–3^ d^–1^ in recovery (range: 0.08–0.15 μg mm^–3^ d^–1^). These means produced a significant RM-ANOVA (*p* = 0.036), with no significant *p*-value between baseline and cold stress (Tukey’s *p* = 0.136), and a significant *p*-value between cold stress and recovery (Tukey’s *p* = 0.0549) (Supplementary Figure S7).

To examine the long-term effects of cool temperature on cellular microcystin concentration, we maintained cultures at 19°C for up to 42 days in two chemostats (81 and 161 μM N). Across nitrate concentrations, the long-term mean microcystin concentration at 19°C was 802 ag μm^–3^ for days 31–42, and was not different than the mean of 850 ag μm^–3^ from days 7–9 (*p* = 0.84) (Supplementary Figure S8).

To test reproducibility, we repeated in biological triplicate the core study described above, but with nitrate concentration set at 161 μM in all chemostats. The temporal patterns in cell concentration, cell size, and cellular microcystin concentration were reproducible (Supplementary Figure S9).

### Decrease in Temperature Induces a General Stress Response

To examine temporal responses in gene expression, we sequenced RNA from 12 samples from a single chemostat over the 21-day experiment. RNA samples were collected from cultures grown in 81 μM N. Sequenced samples were distributed across each of the three experimental periods (Supplementary Figure S10). Sequencing produced a total of ∼131.6 M raw reads, or ∼11 M reads per sample. Collectively, ∼10.1 M reads remained (∼7.7% of total) after *in silico* reduction of rRNA. After trimming for quality, an average of ∼700k reads (or ∼350k cDNA fragments) per sample mapped to the PCC 7806SL reference genome, representing an effective sequencing depth of ∼10.2 fold. Mapping statistics per sample are summarized in Supplementary Table S1.

**FIGURE 3:**
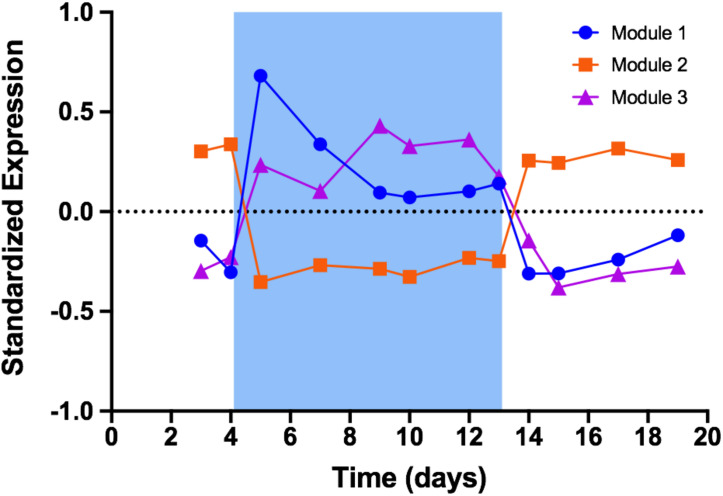
Standardized expression profiles of eigengenes from WGCNA modules. Blue shading represents time period when temperature was set to 19°C. Module 1 represents 673 genes that were quickly and sharply upregulated during cold stress. Module 2 represents 441 genes that were downregulated during cold stress. Module 3 represents 379 genes that were upregulated during cold stress but on a timescale distinctly different from that of module 1.

We used weighted gene correlation network analysis (WGCNA) to analyze patterns of gene expression. In the context WGCNA, a module is a group of genes whose expression is highly correlated among samples, while eigengenes represent the typical expression profile of genes in a given module (Zhang and Horvath, 2005; Langfelder and Horvath, 2008). In PCC 7806, WGCNA identified three modules whose expression responded to changes in treatment temperature (Figure 3; a list of all genes in each of these three modules is provided in Supplementary Table S2). Module 1 (673 genes) and module 3 (379 genes) comprise genes that were upregulated during cold stress. Expression of genes in module 1 was immediately and sharply induced by cool temperature with peak expression occurring within 24 h of the decrease to 19°C. Expression declined through the remaining period of cold stress, then dropped rapidly upon return to 26°C. Module 3 exhibited an immediate but more gradual increase in expression and reached a peak midway through the 19°C period, then dropped rapidly upon return to 26°C. Module 2 (441 genes) comprised genes that were downregulated during cold stress. In this module, expression dropped sharply within 24 h of the decrease to 19°C and remained constant until return to 26°C, whereupon expression rapidly returned to baseline levels.

To gain insight into genes whose transcription responds to temperature changes, we conducted enrichment analysis of Gene Ontology (GO) terms associated with genes in modules 1 and 3 collectively (those upregulated) and in module 2 (those downregulated). GO terms enriched (FDR *p* < 0.05) in combined modules 1 and 3 are listed in Table 1; only the most specific terms are listed. No GO terms were enriched in module 2. The complete list of enriched GO terms, including parent terms, is provided in Supplementary Table S3.

**TABLE 1:** Enriched gene ontology terms derived from genes in combined Modules 1 and 3.

**Enrichment**	**GO ID**	**GO Term**	**FDR *p*-value**
**Molecular Function**			
Over	GO:0003735	Structural constituent of ribosome	7.2E-29
Over	GO:0019843	rRNA binding	3.6E-15
Over	GO:0005524	ATP binding	1.7E-04
Over	GO:0003899	DNA-directed 5′-3′ RNA polymerase activity	1.0E-03
Over	GO:0016776	Phosphotransferase activity, phosphate group as acceptor	1.2E-03
Over	GO:0000049	tRNA binding	1.6E-03
Over	GO:0004812	Aminoacyl-tRNA ligase activity	1.4E-02
Over	GO:0016887	ATPase activity	1.7E-02
Over	GO:0005261	Cation channel activity	3.3E-02
Over	GO:0015450	P-P-bond-hydrolysis-driven protein transmembrane transporter activity	4.7E-02
Under	GO:0004803	Transposase activity	1.0E-02
**Biological Process**			
Over	GO:0009152	Purine ribonucleotide biosynthetic process	1.3E-03
Over	GO:0006418	tRNA aminoacylation for protein translation	9.7E-03
Over	GO:0042254	Ribosome biogenesis	2.3E-02
Over	GO:0006811	Ion transport	2.3E-02
Over	GO:0009206	Purine ribonucleoside triphosphate biosynthetic process	3.3E-02
Over	GO:1901606	Alpha-amino acid catabolic process	4.3E-02
Over	GO:0034470	ncRNA processing	4.6E-02
Over	GO:0043244	Regulation of protein-containing complex disassembly	4.7E-02
Over	GO:0043952	Protein transport by the Sec complex	4.7E-02
Under	GO:0006313	Transposition, DNA-mediated	1.0E-02
**Cellular Component**			
Over	GO:0015934	Large ribosomal subunit	6.7E-05
Over	GO:0045261	Proton-transporting ATP synthase complex, catalytic core F(1)	1.4E-02
Over	GO:0022626	Cytosolic ribosome	1.4E-02
Over	GO:0015935	Small ribosomal subunit	1.5E-02

*This list is reduced to the most specific GO terms only; more general GO terms are removed. FDR *p*-value is the false discovery rate adjusted *p*-value.*

The most striking transcriptional response was an upregulation of almost all genes responsible for ribosomal biogenesis upon decrease in temperature. Genes encoding 52 of 54 ribosomal proteins were upregulated. Genes for ribosomal proteins S7 and S12, which are adjacent in the genome, were the only genes failing to respond to cool temperature. Ten of 23 over-enriched GO terms were directly related to translation.

Cyanobacteria acclimate to cooler temperature by decreasing saturation of fatty acids in glycerolipid membranes (Murata and Wada, 1995; Los et al., 1997). Expression of all primary acyl-lipid desaturases in PCC 7806 were induced by decrease to 19°C, as would be predicted. Genes induced by a decrease in temperature included those responding to oxidative stress (super oxide dismutase *sodB*, peroxiredoxin *ahpC*, thioredoxin and thioredoxin reductase *trxAB*), photosystem II core (*psbA*) and repair proteins (*ftsH2*), and those that respond to more general stress conditions (*rpoAB*, *groEL*, *groES*, *hspA*, and *clpB*) (Drath et al., 2008; Sinetova and Los, 2016). Additional genes upregulated by cool temperature included those involved in transcriptional (*nusG*) and post-transcriptional regulation (homologs of *rbp1* and *rbp2*) and in re-initiation of translation (*hflX*) (Suzuki et al., 2001; Tan et al., 2011; Zhang et al., 2015). Collectively, the gene expression patterns suggest that reduction of temperature to 19°C induces a typical cold-stress response.

### Cellular Microcystin Is Time-Lag Correlated With Expression of *mcy* Genes

**FIGURE 4:**
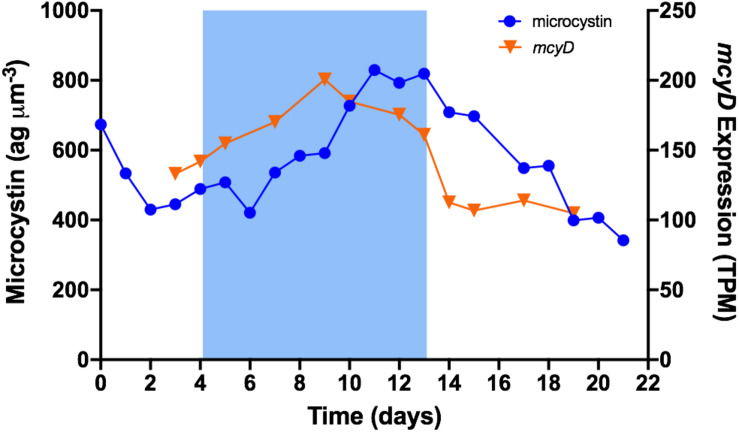
Time trace of *mcyD* expression and normalized cellular microcystin content. Blue shading represents time period when temperature was set to 19°C.

The *mcy* gene cluster encodes enzymes that synthesize microcystin (Tillett et al., 2000). In this study, transcripts were detected for all 10 *mcy* genes. The most highly transcribed across all samples was *mcyJ* with a mean of ∼300 TPM; *mcyC* had the lowest transcription with a mean of ∼57 TPM. Except for *mcyC*, the expression of all *mcy* genes was induced by a decrease in temperature to 19°C; all were more highly expressed during cold stress than in baseline or recovery. *mcyA* grouped in module 1 (those up regulated most rapidly); all others, except *mcyC*, grouped in to module 3 (those increasing more gradually). Figure 4 illustrates the expression of *mcyD* overlaid with the time trace of microcystin content. Expression increased at the onset of cold stress, reached a peak after 5 days at 19°C, started a gradual decline through the remainder of cold stress, then dropped more sharply after the temperature returned to 26°C. Mean expression during cold stress was ∼174 TPM, representing a ∼47% increase over the mean of baseline/recovery (∼119 TPM).

**FIGURE 5:**
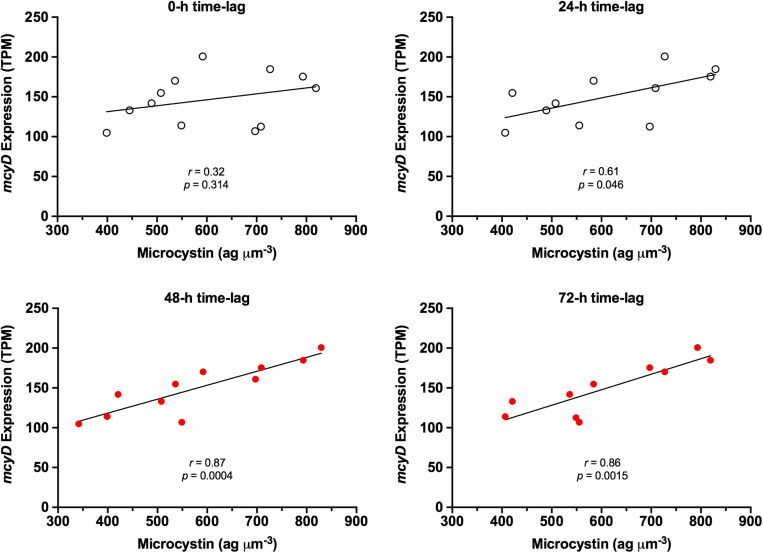
Time-lag effect on correlation between *mcyD* expression and normalized cellular microcystin content. Red dots represent time-lag periods with high correlation and significant *p*-value.

*mcy* gene expression and concurrent microcystin content were not correlated. However, as illustrated in Figure 4, it appears that microcystin content follows a pattern similar to that of *mcyD* expression, albeit with a notable time lag. Time-lag correlation analysis (Schmitt et al., 2004) demonstrates that microcystin content is highly correlated to *mcy* gene expression at a 48-h delay. Using the *mcyD* example, correlation increased from *r* = 0.32 (*p* = 0.314) at 0-h time lag to *r* = 0.87 (*p* = 0.0004) at 48-h time lag, and extended to *r* = 0.86 (*p* = 0.002) at 72 h (Figure 5). All *mcy* genes except *mcyCF* were significantly correlated to cellular microcystin concentration at both 48- and 72-h time lags (range of significant correlation, *r* = 0.66–0.88). *mcyD* was the most highly correlated at 48-h while *mcyE* was the most highly correlated at 72-h. Time-lag correlations of all *mcy* genes are listed in Supplementary Table S4.

### Potential Reference Genes for Use in Experiments With Changing Temperature

The study design allowed us to identify genes whose expression was stable across changing temperatures and thus might potentially serve as reference (housekeeping) genes for use in experiments that include changes in temperature. Genes with an average expression across all time points of >250 TPM and a coefficient of variation < 10% are listed in Supplementary Table S5.

## Discussion

A clear understanding of the environmental cues that govern microcystin production is necessary to enable predictions of bloom toxicity. In addition, a grasp of the mechanisms through which microcystin production is regulated opens the way for agent-based models to predict production at the cellular level by integrating the effects of these cues. With this long-term goal in mind, our objective here was to extend our understanding of how changes in the fundamental parameter of temperature affect the production of microcystin and the resulting quota. The influence of temperature on toxin production in cyanobacteria has been examined frequently [see Supplementary Table S1 in Peng et al. (2018) for a summary of these reports]. In many of those studies, efforts focused on the effects of a change in temperature to one above the optimum for growth, or to one above *in situ* conditions, primarily due to interest in the effects of climate change. The focus of this work is on episodic drops in temperature, defined here as a short-term decrease in temperature to one distinctly below that needed for optimum growth, but which is within the range of temperatures that are still relevant to bloom conditions. In earlier work, we demonstrated in batch culture that a seasonally relevant lower temperature increased cellular microcystin content in *M. aeruginosa* NIES-843 (Peng et al., 2018). The current work is a follow-up of that study meant to confirm this response in another strain of *M. aeruginosa*, and to describe both the physiologic and temporal nature of the response to this change in temperature.

We investigated *M. aerugino*sa PCC 7806 because it, along with NIES-843, are the most commonly used lab strains in *Microcystis* research (Harke et al., 2016). We note that these cyanobacteria were originally isolated from disparate geographic locations and have notably different genome sizes (Meyer et al., 2017), and thus represent dissimilar strains. Our observations confirm, in a second strain, an effect of below-optimal temperature on microcystin quota. RNA sequencing identified physiological responses associated with an increase in toxin production, while WGCNA and time-lag analysis revealed metabolic processes operating at different timescales and directly demonstrated a temporal disconnect between *mcy* transcript abundance and cellular toxin content. We frame these observations within the ecology of *Microcystis* and the potential for multiple environmental factors to influence cellular growth and toxin production (Wilhelm et al., 2020).

### Temperature Effect on Cellular Microcystin Concentration

Like NIES-843, PCC 7806 increased microcystin quota when grown at 19°C relative to 26°C, demonstrating that this response to cool temperature is not unique to a single strain. Differences in experimental methods prevent direct comparisons between the two strains, but relative comparisons demonstrate that the response in each strain was similar. In batch cultures of NIES-843, average microcystin quota was ∼2.1 fold higher at 18°C than at 26°C (Peng et al., 2018). In continuous cultures of PCC 7806, cellular microcystin concentration increased ∼1.9 fold at 19°C relative to 26°C.

Under the conditions tested here, nitrate concentration did not meaningfully alter the increase in cellular microcystin content due to cool temperature. This is similar to the earlier findings in batch cultures of NIES-843. It is interesting to note that the cold-induced increase in microcystin content fully manifested itself within ∼8–9 days at 19°C. Long periods at 19°C (31–42 days) did not significantly alter microcystin concentration from that observed after 9 days. This suggests that higher microcystin content is maintained at this temperature and is not a short-term shock response.

Lastly, calculations demonstrated that microcystin production had to increase at 19°C and decrease upon return to 26°C to account for the observed cellular microcystin concentrations. This observation is at odds with the general trends outlined in Long et al. (2001), which indicated that microcystin quota can be predicted from growth rate in nitrogen-limited chemostats.

Calculations of production were based on measurements of particulate (intra-cellular) microcystin. As a result, these production estimates do not account for dissolved microcystin released due to cell death. However, an increase in cell death rates, *e.g.*, in response to a decrease in temperature, results in an increase in the unaccounted-for-loss of microcystin from the chemostat, which necessitates a production rate higher than our calculation. We therefore feel our estimates of production are conservative.

### Transcriptional Response of *Microcystis* to Decrease in Temperature

It has been said that “blooms like it hot” (Paerl and Huisman, 2008). That said, we expected that a 7°C decrease would induce a “cold-stress” response leading to a characteristic transcriptional profile. To test this, we compared the expression profile of PCC 7806 to other cyanobacteria in the literature (*Synechocystis* and *Synechococcus*), as studies of *Microcystis* are limited. In PCC 7806, the most extensive changes were increased ribosomal biogenesis and an upregulation of genes encoding ribosomal proteins. This pattern has been observed in both *Synechococcus* PCC 7942 (Billis et al., 2014) and *Synechocystis* PCC 6803 (Suzuki et al., 2001; Billis et al., 2014; Sinetova and Los, 2016). This response seems counterintuitive when compared to changes observed in model heterotrophic bacteria. For example, in *Escherichia coli*, ribosomal biosynthesis typically increases with change in conditions that stimulate rapid growth, rather than with conditions that slow growth as observed here (Vadia and Levin, 2015). The increase in translational machinery due to cold-stress has been interpreted as a compensatory response necessary to maintain adequate protein synthesis in the cell as the rate of synthesis per unit ribosome deceases at low temperature (Suzuki et al., 2001). Low temperature directly inhibits synthesis of proteins involved in photodamage repair of photosystem II (PSII) (Kanervo et al., 1997; Allakhverdiev and Murata, 2004). Thus, it has been suggested that this compensatory response is important in cyanobacteria as a way to maintain repair of PSII during periods of low temperature (Suzuki et al., 2001). Reports of this pattern in eukaryotic phytoplankton suggest that it could be a more general phenomenon of photosynthetic organisms (Rhee and Gotham, 1981).

A well-documented transcriptional response to cold stress is an upregulation of genes involved in desaturation of fatty acids in membrane lipids. A decrease in saturation is necessary to maintain membrane fluidity at lower temperatures (Murata and Wada, 1995). *Synechocystis* PCC 6803 increased transcription of all desaturase genes within 60 min of a 12°C downshift in temperature (Los et al., 1997). Likewise, in thermotolerant *Synechococcus* PCC 7002, growth at 30°C produced a prominent upregulation of desaturases relative to growth at 38°C (Ludwig and Bryant, 2012). Within 24 h of temperature decrease, transcription of all desaturases in PCC 7806 was sharply increased, consistent with well-described cold-stress responses.

Finally, expression of additional genes, including chaperone proteins commonly associated with heat shock (*groES*, *groEL*, *hspA*), sigma factors (*rpoA*), and PSII core and repair proteins (*psbA*, *ftsH*), were increased by cool temperature in a pattern similar to those in *Synechococcus* and *Synechocystis* (Inaba et al., 2003; Billis et al., 2014; Sinetova and Los, 2016).

Rocca et al. (2015) pointed out that transcriptional studies often assume a positive relationship between transcript abundance and rates of the corresponding process, a relationship that is not always valid. In a study relevant here, in a strain of marine *Synechococcus* responding to cold stress, the transcriptional patterns outlined above were largely confirmed at the point of protein abundance, suggesting a generally conserved and bona fide response in these cyanobacteria (Varkey et al., 2016).

In a recent study, Antosiak et al. (2020) used *Raphidiopsis (Cylindrospermopsis) raciborskii* to examine expression of a subset of genes thought to be associated with the acquired chill-light tolerance response. Though the timing of transcriptional change varied, there was a high degree of correspondence between *Raphidiopsis* and PCC 7806 in genes upregulated by cool temperature. Among these were *desA*, *ftsH*, *hflX*, *nusG*, and *rbp1*. This correspondence between distantly related species suggests a conserved response across cyanobacteria. In contrast to *Raphidiopsis* (Antosiak et al., 2020) and *Synechocystis* PCC6803 (Li et al., 2012), the *ccr2* homolog (cyanobacterial cold resistance) in PCC 7806 did not exhibit notable changes in expression due to decrease in temperature; nor did *ccr1* (Yin et al., 2007). Ccr2 is a thylakoid-associated protein (Li et al., 2012) and microcystin is known to preferentially localize near thylakoids in PCC 7806 (Young et al., 2005). As neither *Raphidiopsis* nor *Synechocystis* produce microcystin, it would be interesting to determine if *ccr2* responds differently in non-toxic strains of *Microcystis*.

In PCC 7806, the expression profile of a group of genes was especially notable. These genes included the sigma factor *rpoB*, the protease *clpB*, the heat shock protein *hspA*, superoxide dismutase *sodB*, the peroxiredoxin *ahpC*, and thioredoxin and thioredoxin reductase *trxAB*, respectively. These genes play a part in maintaining redox balance in the cell and their transcription is known to increase during oxidative stress (Billis et al., 2014; Sinetova and Los, 2016). Consequently, their upregulation in PCC 7806 at 19°C suggests that *Microcystis* experienced oxidative stress in this condition. However, our interpretation on this point was complicated by the fact that transcriptional response within the complement of peroxiredoxins was incomplete. Notably, none of the four peroxiredoxins of the PrxQ-subclass showed increased expression, indicating that this group of putative peroxidases likely responds to oxidative stress stimuli not triggered by a reduction in temperature.

The similarity of gene expression profiles between PCC 7806 and other model cyanobacteria demonstrated that a decrease in temperature induced a classic cold-stress response in PCC 7806. While perhaps obvious, this conclusion remains important as it confirms the physiological status of the cell, places the response of microcystin quota into context, and demonstrated that experimental methods were robust enough to detect predicted responses.

### Cold-Stress Response Is Partitioned Between Short- and Long-Term Time Scales

In continuous cultures of PCC 7806, a decrease in temperature induced *mcy* gene expression, microcystin production and microcystin cellular concentration. Concomitantly, this decrease in temperature also induced a classic cold-stress response that appears to operate on a timescale different from that of microcystin production. The question then arises as to whether these responses are one in the same? In bacteria, transcriptional response to stress typically occurs with amazing speed: in *E. coli*, for example, responses to cell envelope damage can be detected in as little as 3 min (Ades et al., 1999), while robust responses to induced oxidative stress occur within 10 min (Jozefczuk et al., 2010). In *Synechocystis* PCC 6803, exposure to a reduced temperature induces detectable changes in gene expression within 20 min (Los et al., 1997). In this study, the expression of genes in module 1 increased sharply within 24 h of temperature decrease, which was the time resolution of our experiment. In contrast, *mcy* gene expression, along with other genes in module 3, increased over a period of about 5 days while microcystin concentration increased over a period of 7 days. As a stress response, *mcy* expression and microcystin production seem to operate over an unusually protracted period. Given the distinct timescales over which genes in module 1 and 3 seem to function, it is possible these genes responded to different manifestations of the same stress stimuli and belong to different regulatory groups. Taken together, our data hint at a partitioning of the cold-stress response in *Microcystis* between short-term and long-term regulatory networks.

The fact that cellular microcystin concentration increases slowly over a period of days is puzzling. It may be that microcystin biosynthesis is finely calibrated to the damage it presumably mitigates, and that this injury, which can be caused by cool temperature, accumulates incrementally over days. Consistent with these findings, the timescale over which microcystin quotas change in this experiment is similar to that shown in continuous cultures responding to changes in light (Utkilen and Gjølme, 1992). Collectively, these studies support the concept of long-term stress responses and regulatory networks. Unfortunately few studies designed to capture the long-term dynamic response to cold-stress in cyanobacteria are available for comparison. Moreover, the many studies comparing batch cultures grown at different yet constant temperatures do not capture the dynamic nature of the response; they instead observe physiology acclimated to specific temperatures. Additional work investigating various timescales will be needed to clarify and confirm this conjecture.

### Change in Cell Size

The increase in cell size that occurred during lower temperature is consistent with indications of a generalized stress response. We base this conclusion tentatively on studies demonstrating that cell size in *M. aeruginosa* is generally inversely related to growth rate (Long et al., 2001; Yeung et al., 2016) and increases under either general stress conditions (Krüger and Eloff, 1981) or during growth at cooler temperatures (Chen et al., 2011). Our results are consistent with these findings and hint at a more general relationship between cell size and stress in *M. aeruginosa*. An exception to this trend comes from Yin et al. (2016). These authors found that cell size in *M. aeruginosa* increased with increasing temperature. It is possible that strain variation (they do not report the specific isolate they used) or specific experimental conditions (they grew strains under 12:12 day:night cycles and in batch culture) may exert enough influence to alter what might be a general trend.

The inverse relationship between cell size and nitrogen concentration was surprising, as it seems to contradict the surface area to volume principle that permits greater nutrient flux per unit cell volume in smaller cells (Grover, 1989; Chisholm, 1992). While data are somewhat scarce, other cyanobacteria seem to follow the surface area to volume concept under some observed nutrient gradients. For example, in *Synechococcus* and *Anabaena flos-aquae*, cell size was notably reduced in iron-limited cultures (Gorham et al., 1964; Sherman and Sherman, 1983) and similar observations have been made with natural communities in the Pacific Ocean (Eldridge et al., 2004; Hare et al., 2005). Nevertheless, if a phenomenon exists whereby stress induces an increase in cell size in *Microcystis*, it is possible that the inverse relationship between cell size and nitrogen concentration is a result of N-limited stress superseding any advantages gained by more efficient nutrient flux due to smaller cells.

The precise mechanism regulating cell size in bacteria is still debated (Harris and Theriot, 2018). Intriguingly, our preliminary observations in *M. aeruginosa* seem in agreement with the relative rates model proposed by Harris and Theriot (2016) in which cell size is governed by the relative increase in cell envelope biomaterial *vs.* increase in cell volume. These authors further posited that increase in surface area material is likely regulated by peptidoglycan synthesis. In our case, expression of *glmS*, the enzyme responsible for the first committed step in peptidoglycan synthesis (Barreteau et al., 2008), dropped sharply when the temperature decreased to 19°C, generally rose slowly through the remainder of cool period, then increased quickly when the temperature returned to 26°C. Based on the relative rates model, this pattern could, in principle, explain the increase in cell size during cold stress and a decrease when warm temperature returns. Likewise, it is possible that other stressors exert their influence and increase cell size by suppressing cell envelope synthesis. While cell size is known to influence key physiological processes in phytoplankton, this subject is outside the purview of this study. We report on it here only because it affects normalization methods, it is consistent with other reports of stress response, and it may provide information useful to other researchers.

### Proposed Mechanism for Microcystin Response to Cold Stress

The mechanism responsible for increased synthesis of microcystin during cold stress is uncertain. In a widely cited paper, Zilliges et al. (2011) hypothesized that microcystin mitigates the effects of oxidative stress on Calvin cycle proteins. Building upon this hypothesis, we suggest a provisional mechanism for the stimulation of production of microcystin during cool temperatures. Conceptually, the mechanism would be activated in the following way. A downshift in temperature is known to suppress photodamage repair of PSII (Allakhverdiev and Murata, 2004; Murata et al., 2007), leaving the reaction center in a decreased state of repair after a period of lower temperatures. Compounding this strain, under constant light, a decrease in temperature increases PSII excitation pressure and offsets cellular redox balance *via* the enzyme-mediated transport chain (Maxwell et al., 1995). In combination, these factors trigger an oxidative stress response, of which an increase in microcystin production is part.

The proposed mechanism is compatible with results by Kaebernick et al. (2000) in which PCC 7806 exposed to high light intensity increased transcription of *mcyBD*, but did not increase detectable cellular microcystin content. The group later demonstrated that high light intensity and oxidative stress stimulated binding of microcystin to photosynthetic proteins, thus linking microcystin to an oxidative stress response and at least partially explaining the apparent disconnect between expression of *mcy* and quota (Zilliges et al., 2011; Meissner et al., 2013). Excitation pressure in PSII increases with light intensity if temperature is constant and increases at constant light if temperature is decreased (Maxwell et al., 1995). Consequently the two methods should, in principal, lead to an equivalent or similar modulation of excitation pressure (Maxwell et al., 1995). In this way, the observed responses to cold stress *vs.* high light may be one in the same.

We see an increase in *mcy* expression with a concomitant increase in cellular microcystin content over time. But this increase in content occurs only after a small, short-term decline 24–48 h after the downshift in temperature. This temporary decline is consistent with the concept that induction of oxidative stress stimulates microcystin binding to proteins, thus hiding that portion of the cellular pool of microcystin from standard detection methods (Meissner et al., 2013). Moreover, the timescale over which it occurs coincides with that demonstrated in Schuurmans et al. (2018). These authors hypothesized that the short-term decline was due to binding of microcystin to proteins upon induction of severe oxidative stress *via* application of hydrogen peroxide.

### Time-Lag Correlation of Microcystin and *mcy* Expression

Several studies have examined the relationship between *mcy* expression and microcystin quota, and found either no or (at best) a weak correlation (Kaebernick et al., 2000; Tonk et al., 2005; Sevilla et al., 2008; Peng et al., 2018). This might be explained in part by the fact that environmental stimuli that induce *mcy* transcription can also stimulate the binding of microcystin to cellular proteins, thus hiding this portion of microcystin from detection (Meissner et al., 2013). Our results provide an additional potential explanation.

Under the conditions tested, we also saw no correlation between *mcy* expression and concurrent microcystin concentration, but instead observed a very strong 48 h time-lag correlation of microcystin content to *mcy* expression. The reason for such a long time lag between gene expression and the manifestation of that expression is unclear. The relationship could be an artifact of experimental conditions such as strain specificity, continuous irradiance, dilution rate, or experimental duration. But the fact that *mcy* expression was highly time-lagged correlated to microcystin concentration over a 21-day period across conditions of both increasing and decreasing microcystin production is intriguing and has potentially exciting implications. If this observation can be confirmed in other strains, other experimental designs, and in field conditions, it opens a future possibility of forecasting fluctuations in bloom toxicity with a 48- to 72-h lead-time.

## Conclusion

In this work, we demonstrated that cellular microcystin concentration increases when *M. aeruginosa* PCC 7806 is subjected to temperatures below those optimal for growth, confirming this phenotype in a separate disparate strain. Gene expression profiles demonstrated that the drop in temperature induced a cold-stress response in *Microcystis* but suggested that the increase in microcystin production may be regulated through separate transcriptional networks and on a different timescale. This simple method of manipulating microcystin production in the laboratory should prove valuable in exploring the eco-physiological function of microcystin. This response to cool temperature has potential to partially explain the observation that natural blooms are frequently found to be more toxic during late spring when water temperature is cooler and less toxic in mid-summer as water temperatures rise. Similarly, the work suggests that episodic climactic events that can cause significant reduction in surface temperature might influence toxin production by *in situ* populations. We show that under the conditions tested, microcystin concentration is highly time-lag correlated to *mcy* gene expression. If confirmed under field conditions, this raises the possibility of estimating fluctuations in bloom toxicity with a 48-h lead-time by monitoring gene expression.

## Data Availability Statement

The datasets presented in this study can be found in online repositories. The names of the repository/repositories and accession number(s) can be found in the article/Supplementary Material.

## Author Contributions

RM, SW, FH, and AP conceived and designed the research. RM and GS performed the experiments. DD, BW, and GB performed the microcystin analysis. RM, MM, and EG analyzed the data. RM and SW wrote the draft manuscript. All the authors contributed to and reviewed the final version of the manuscript.

## Conflict of Interest

The authors declare that the research was conducted in the absence of any commercial or financial relationships that could be construed as a potential conflict of interest.
