# Supplementary material for: Episodic Decrease in Temperature Increases mcy Gene Transcription and Cellular Microcystin in Continuous Cultures of Microcystis aeruginosa PCC 7806
Source: Front Microbiol. 2020 Dec 3;11:601864. doi: 10.3389/fmicb.2020.601864 (PMC7744600; doi:10.3389/fmicb.2020.601864)
Supplement: Supplementary file 6 [file Data_Sheet_1.pdf]

Supplementary Material

1 Supplementary Figures and Tables

1.1 Supplementary Figures

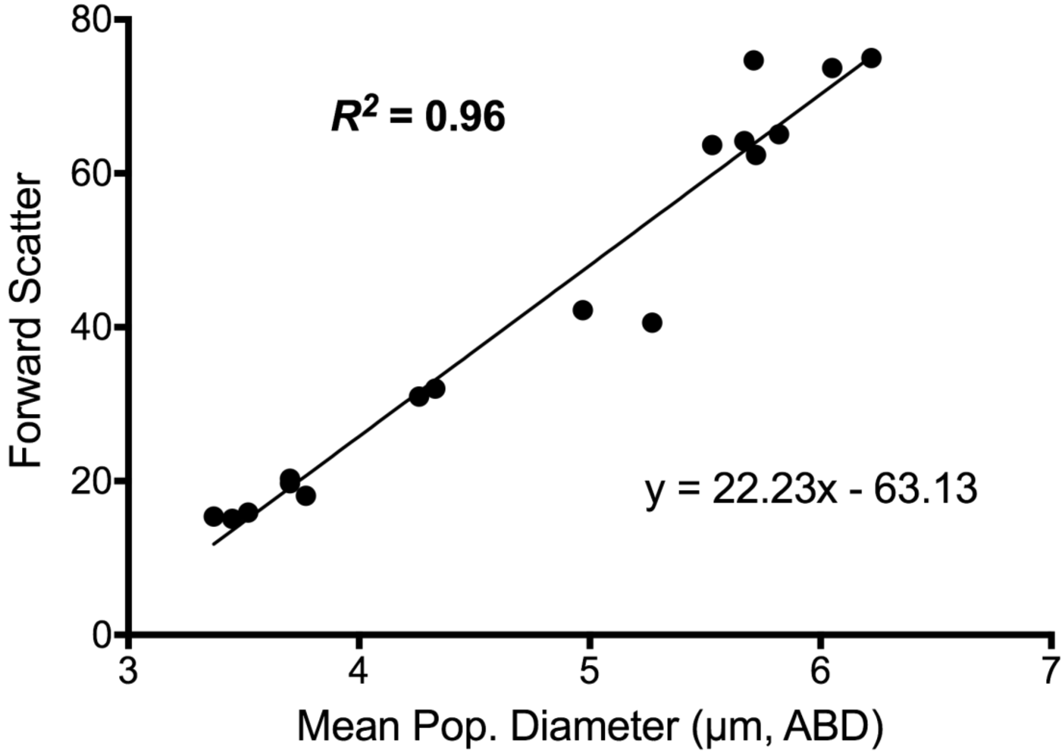

Supplementary Figure 1. Relationship between forward scatter (from flow cytometry using Guava easyCyteHT) and population mean diameter in cells of *M. aeruginosa*. Equation from linear regression.

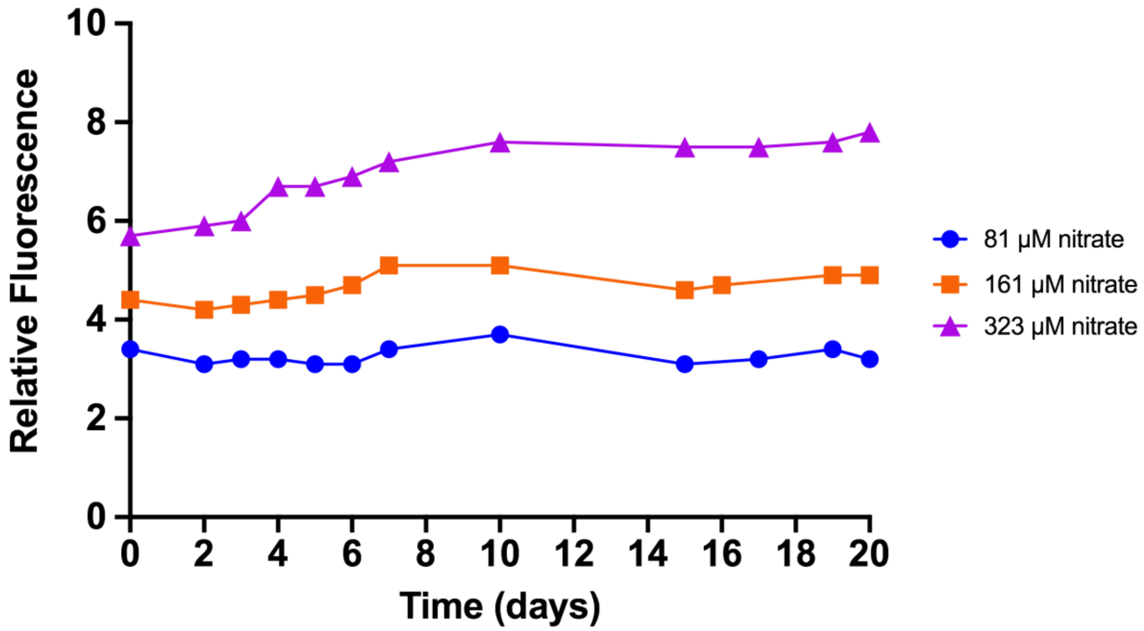

Supplementary Figure 2. Relative fluorescence of individual cells of *M. aeruginosa* PCC 7860 grown in different concentrations of nitrate over a 20-d period at steady state. Fluorescence was measured *via* flow cytometry.

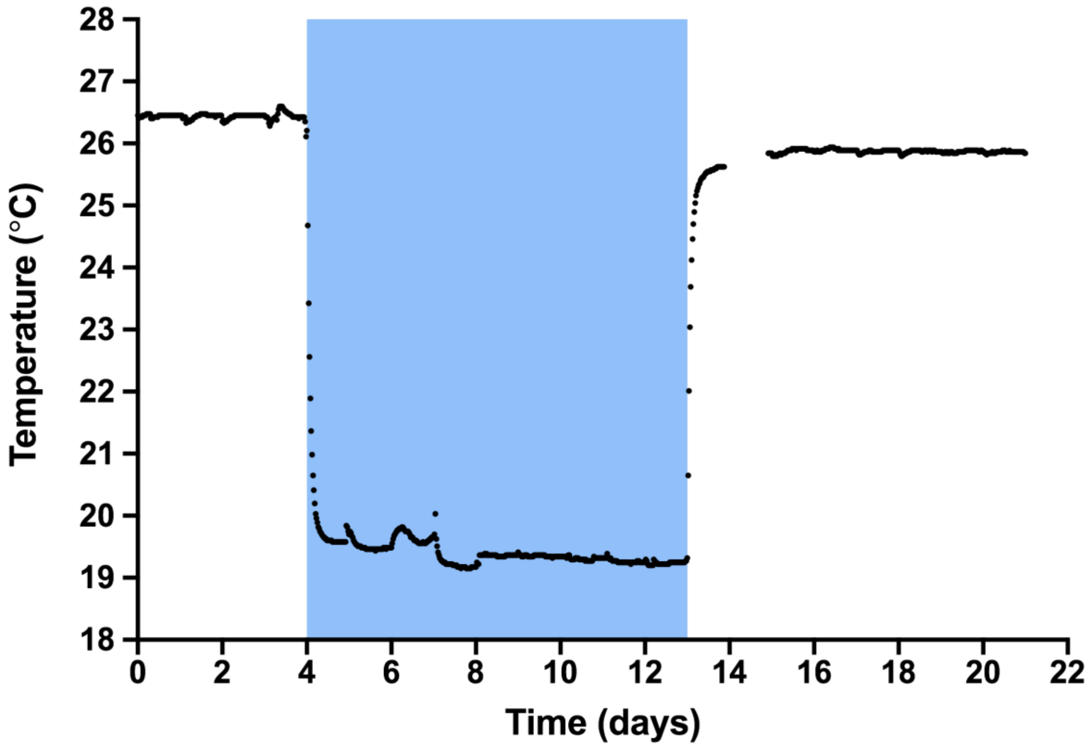

Supplementary Figure 3. Temperature of culture media during experimental period. Temperature was measured every 30 min and a point is plotted for each measurement. Temperature data logger malfunctioned for a 24.5 h period around day 14 which can be seen as an absence of data for that period.

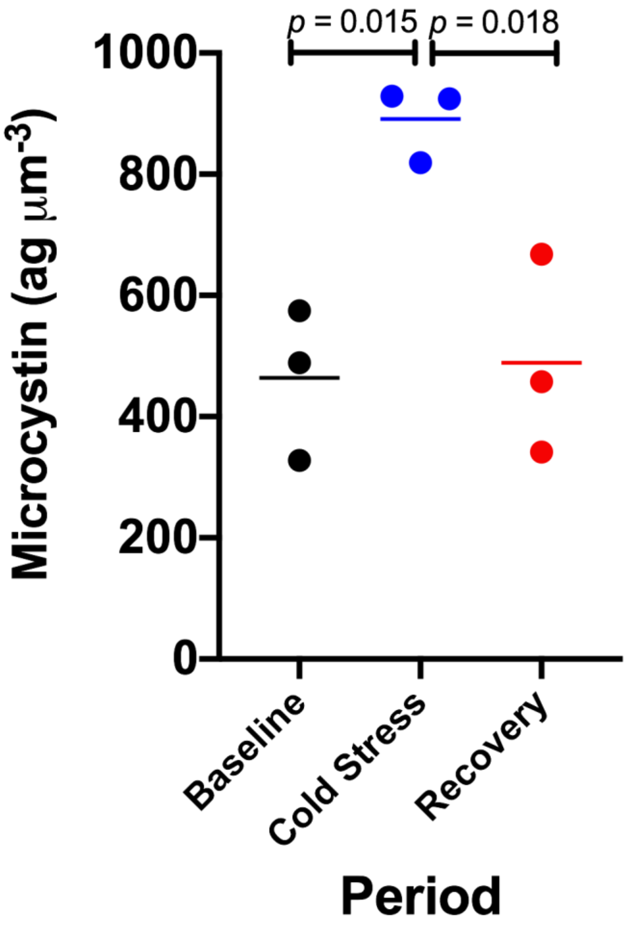

Supplementary Figure 4. Mean cellular microcystin content on the final day of each experimental temperature period. Reported *p*-values from RM-ANOVA.

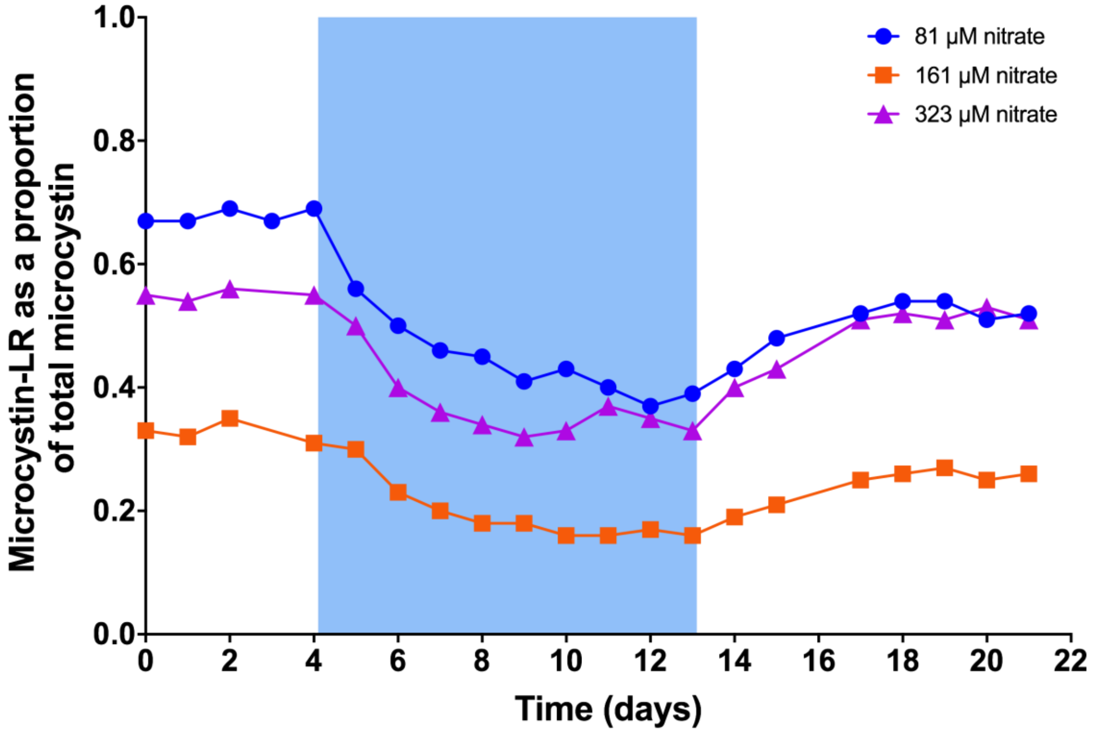

Supplementary Figure 5. Profile of detected microcystin congeners across experimental time. Two congeners were detected: microcystin-LR and [D-Asp3]-microcystin-LR. The figure illustrates microcystin-LR as a proportion of total microcystin.

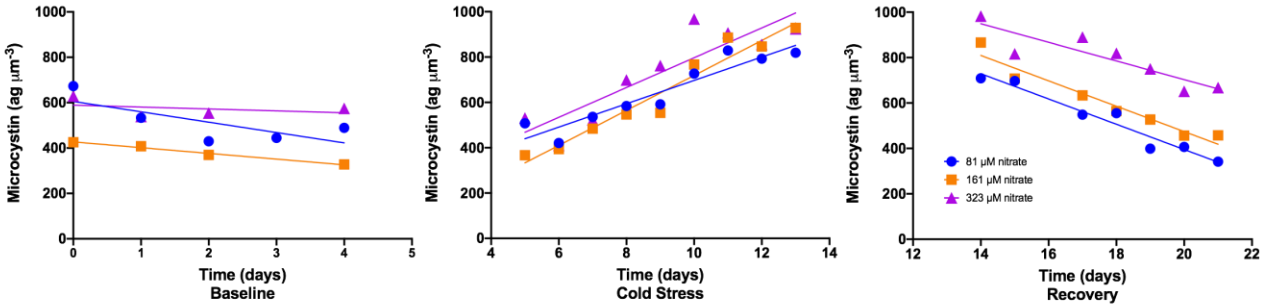

Supplementary Figure 6A. Relationship of cellular microcystin content to time for each temperature period: baseline, 26° C; cold stress, 19° C; recovery 26° C. Slopes for each N concentration:period combination was calculated by linear regression.

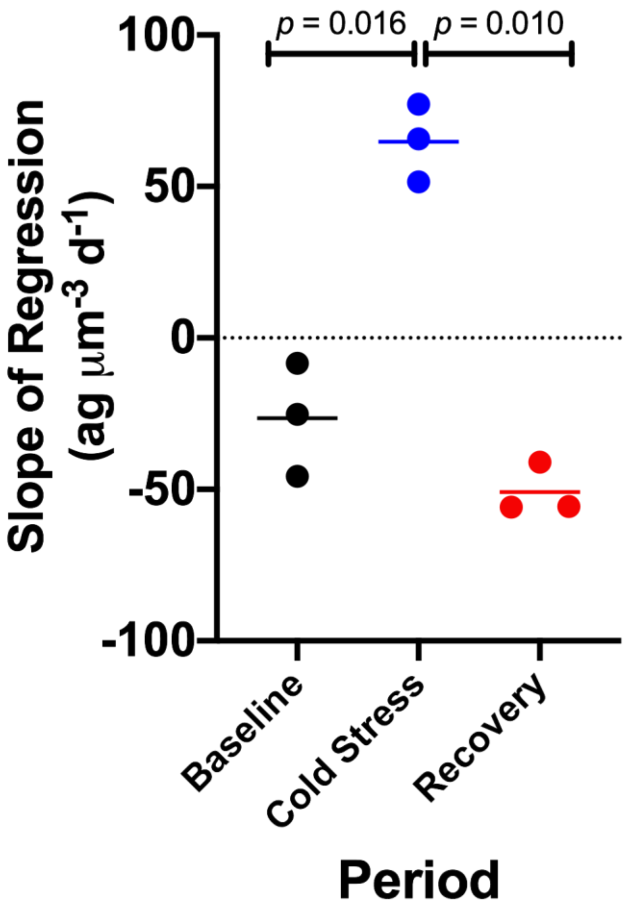

Supplementary Figure 6B. Relationship of cellular microcystin content to time for each temperature period: baseline, 26° C; cold stress, 19° C; recovery 26° C. Summary of slopes for each N concentration:period combination and RM-ANOVA results.

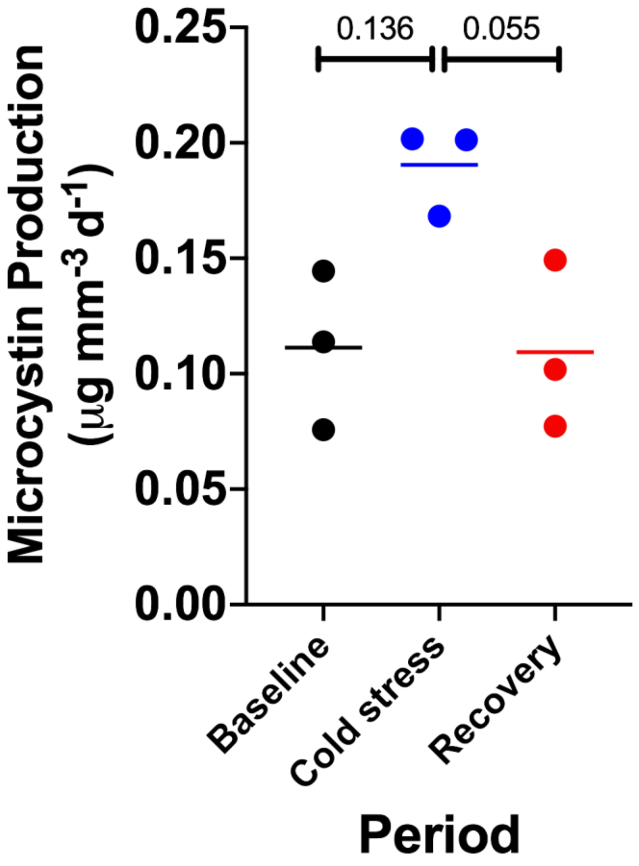

Supplementary Figure 7. Microcystin production per unit biovolume by experimental time period. Reported *p*-values from RM-ANOVA.

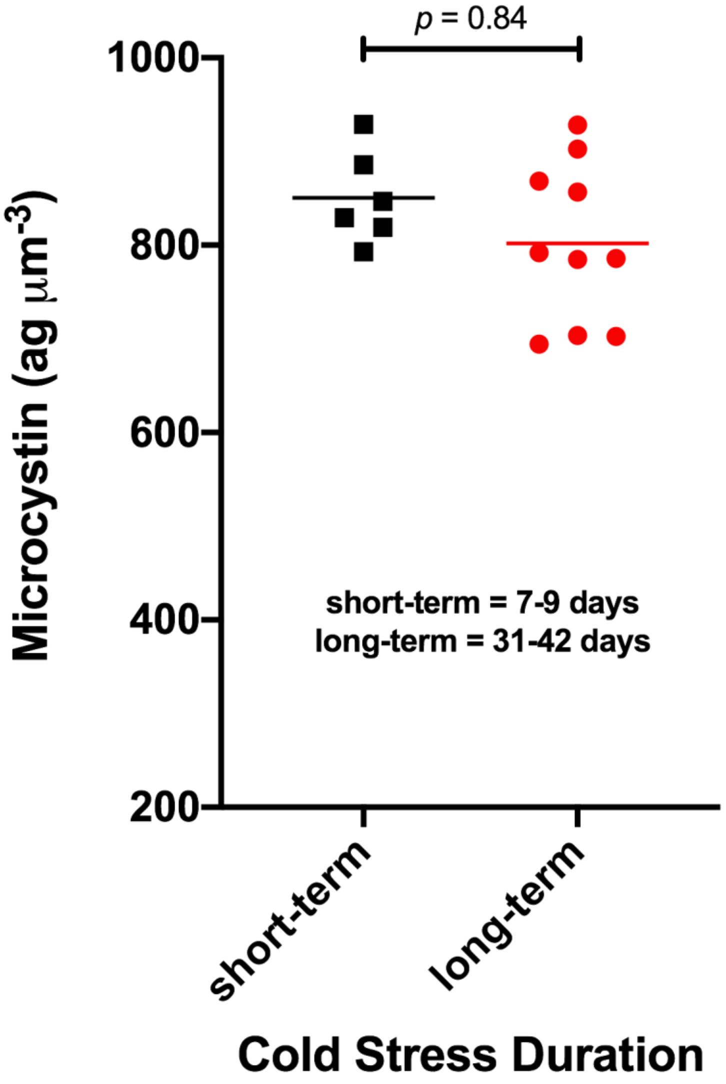

Supplementary Figure 8. Comparison of microcystin quota in *M. aeruginosa* PCC 7806 after short-term (7-9 days) vs. long-term (31-42 days) cold stress (19° C). Observations from cultures grown in 81 and 161 µM nitrate are combined.

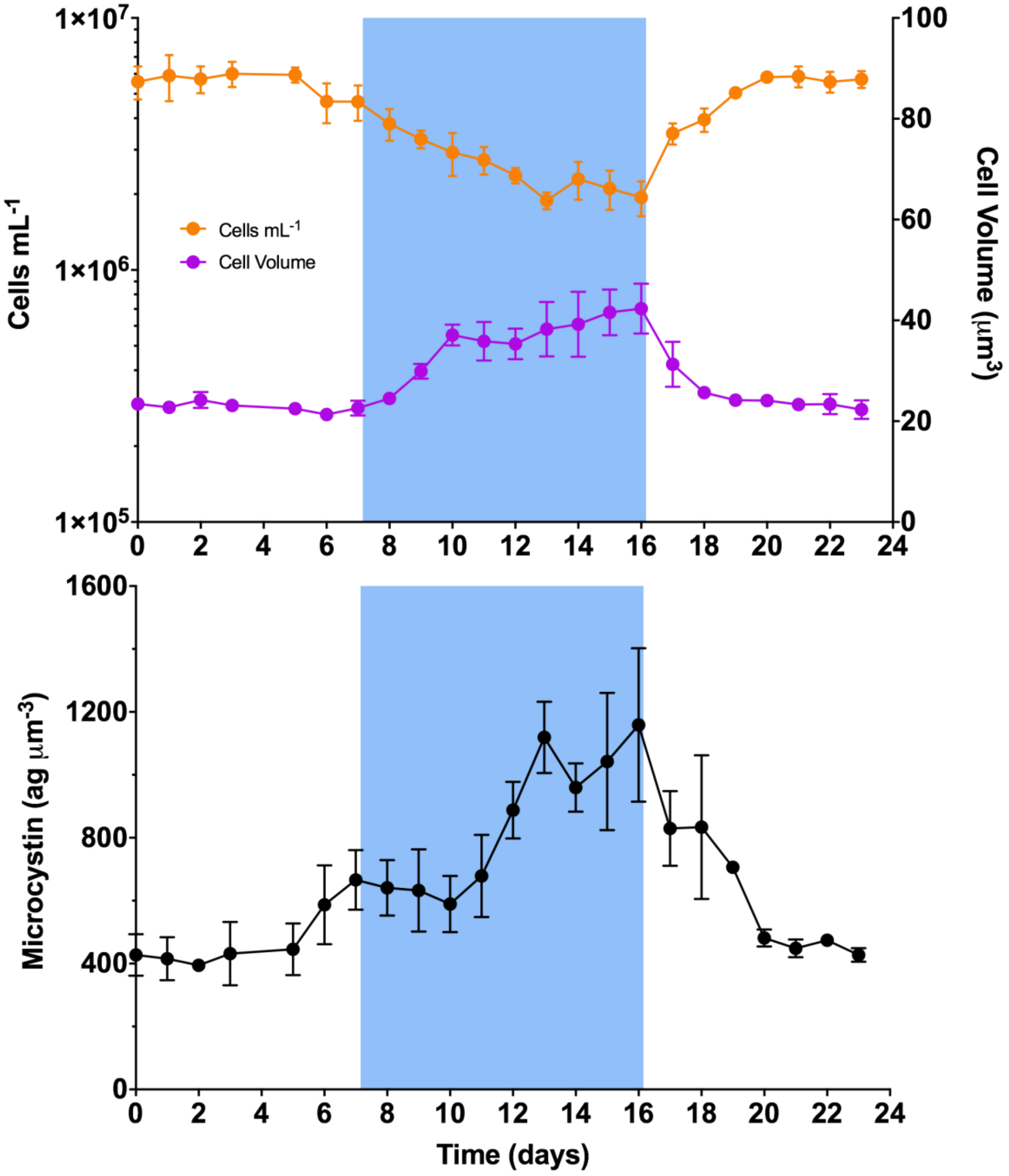

Supplementary Figure 9. Reproducibility of experimental results. (see text). Dynamics of cell concentration, cell volume, and microcystin quota in continuous cultures of *M. aeruginosa* PCC7806 over experimental time. Blue shading represents the time period when temperature was set to 19° C; other periods temperature was 26° C. All cultures grown in 161 µM nitrate. n = 3.

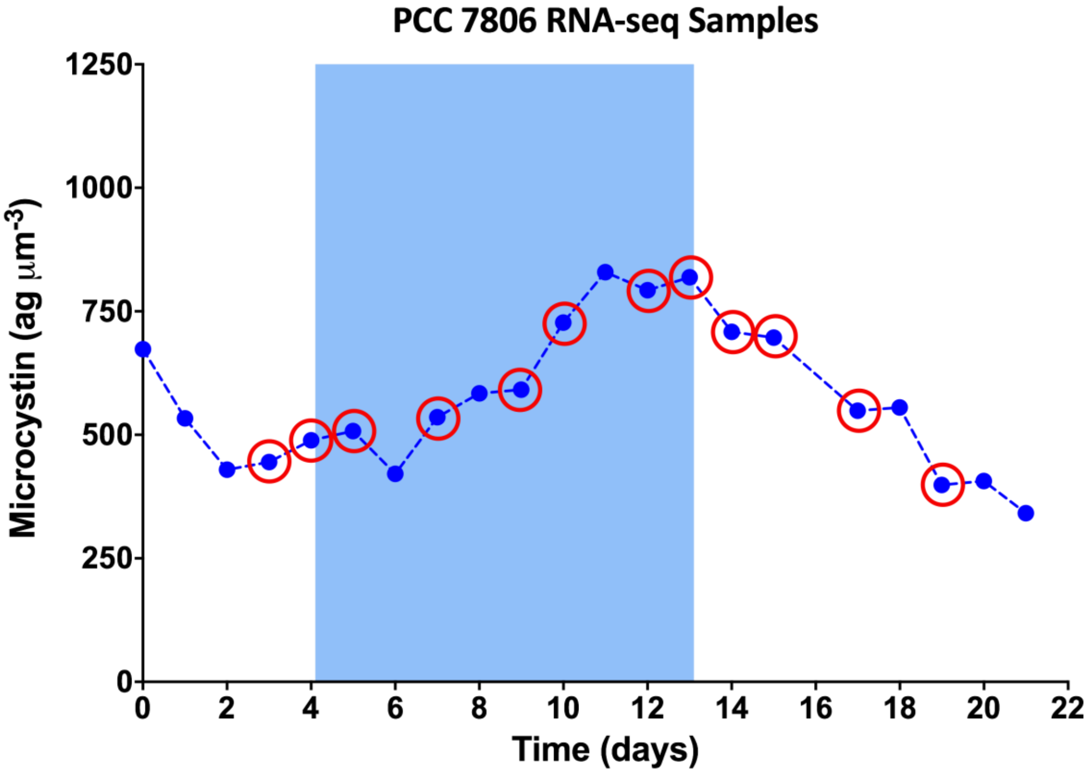

Supplementary Figure 10. Experimental time trace of *M. aeruginosa* PCC7806 growing in 81 μM nitrate. Blue dots represent sampling points. Red circles indicate sample points for which RNA was sequenced.
